# A method for estimating energy parameters of RNAs by differentiating base-pairing probabilities

**DOI:** 10.1093/nargab/lqaf171

**Published:** 2025-12-08

**Authors:** Kazuteru Yamamura, Goro Terai, Kiyoshi Asai

**Affiliations:** Department of Computational Biology and Medical Sciences, Graduate School of Frontier Sciences, University of Tokyo, Kashiwanoha 5-1-5, Kashiwa, Chiba 277-8561, Japan; Department of Computational Biology and Medical Sciences, Graduate School of Frontier Sciences, University of Tokyo, Kashiwanoha 5-1-5, Kashiwa, Chiba 277-8561, Japan; Department of Computational Biology and Medical Sciences, Graduate School of Frontier Sciences, University of Tokyo, Kashiwanoha 5-1-5, Kashiwa, Chiba 277-8561, Japan

## Abstract

The structure of RNA is deeply related to its function, and information about RNA substructure energy parameters is useful for predicting its structure from its sequence. RNA in cells is often modified, and these various types of modifications affect its structure and function. In recent years, the use of pseudouridine modifications in RNA vaccines has increased the importance of predicting structures that include modified bases. However, energy parameters of substructures involving modified bases have not yet been sufficiently determined. Therefore, in this paper, we propose a method for inversely calculating energy parameters from base-pairing probabilities. This method optimizes energy parameters using the same mechanism as gradient descent in deep learning. We also propose efficient computational approaches, including the calculation of the derivative of the partition function using a dynamic programming method following computations with the McCaskill algorithm. Because base-pairing probabilities can be obtained by adjusting them through chemical probing methods, it is expected that parameter estimation can be performed without relying on labor-intensive experiments or molecular dynamics simulations.

## Introduction

RNA undergoes various types of chemical modifications within the cell [1], some of which are known to alter base-pair formation and structural stability [2–4]. To predict RNA structures, the Zuker algorithm, which identifies the minimum free energy (MFE) structure, is commonly used [5]. In addition, the McCaskill algorithm is employed to compute the base-pairing probability of each base [6], and these calculations require energy parameters for various RNA substructures, such as stacks, bulges, and hairpins [7, 8]. Predicting the structure of RNA molecules containing modified bases requires appropriate energy parameters for those modifications. The importance of modified nucleotides has been increasingly recognized in recent years. It has been shown that various chemically modified RNAs do not trigger innate immune responses in mammals [9]. In addition, the use of pseudouridine in messenger RNA (mRNA) has been demonstrated to produce non-immunogenic vectors [10]. These findings have accelerated the practical application of mRNA vaccines. Although such medical applications have been realized, it is also well known that the RNA structure of mRNA vaccines plays a critical role in their thermodynamic stability and translational efficiency [11]. Therefore, understanding RNA structures that include modified bases remains an essential and pressing challenge. Some of the energy parameters for modified bases, mainly stacking parameters, have been determined through biological experiments and molecular dynamics simulations [12–19], and it is possible to calculate the secondary structure and base-pairing probabilities of RNA, including modified bases, using these empirically determined and computationally estimated parameters [20]. Our group has also developed a method that combines molecular dynamics simulations and experimental data [19]. Using this method, we estimated the energy parameters of inosine-modified RNA. However, both experimental and simulation-based approaches are labor-intensive, and it is difficult to determine a sufficient number of energy parameters for modified bases using these approaches.

In recent years, structure prediction methods using deep learning have also been developed [21]. Some of these new deep learning-based models do not rely on energy parameters, but predicting novel RNA families that are not included in training data remains a challenge [22], and hybrid approaches such as MXfold2 [23], which combines thermodynamic energy parameters and machine learning techniques, have been suggested as alternative approaches [22]. In addition to structure prediction, mRNA design has also been actively studied [24]. While many deep learning-based methods have been proposed for this design task, highly performing approaches that depend on energy parameters are still being actively developed [25–27]. Thus, despite the current widespread use of machine learning-based methods, thermodynamic parameters remain highly important in RNA structure prediction and design.

**Figure 1.**
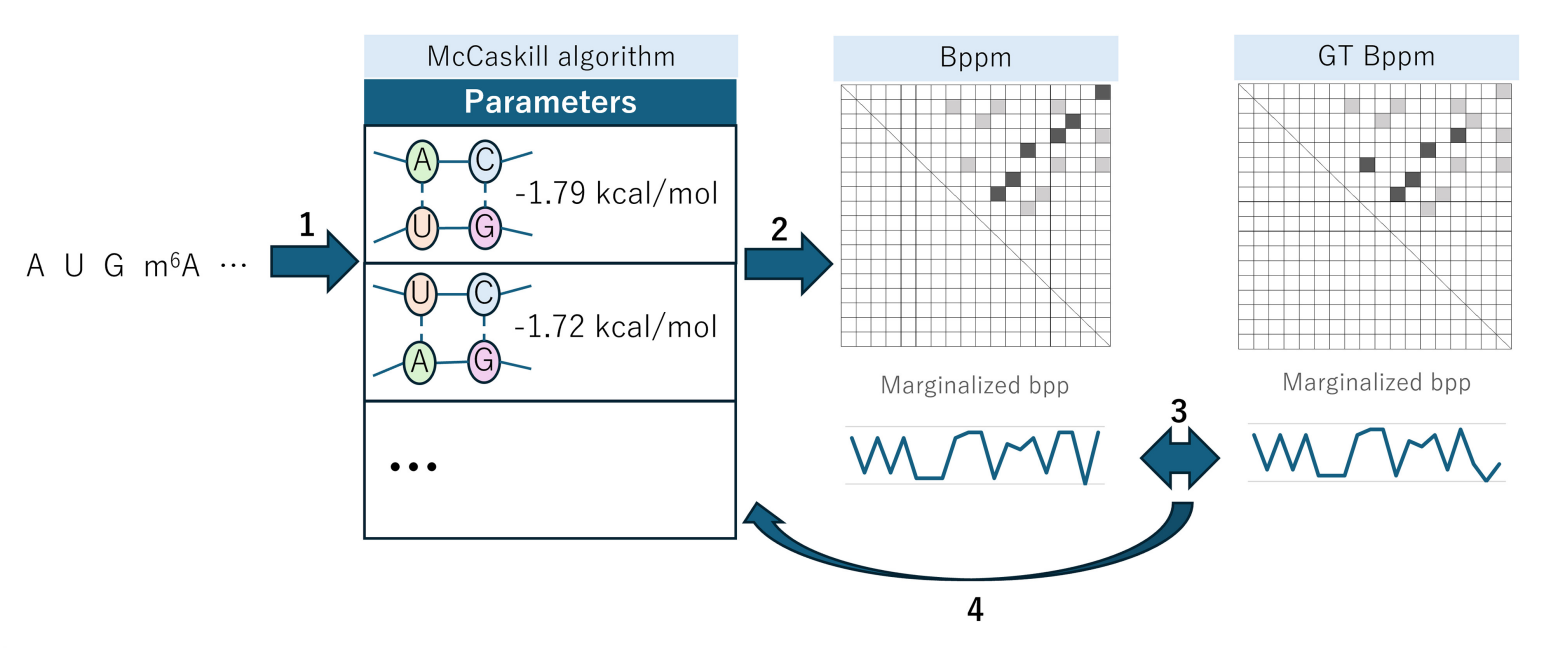
Conceptual diagram of the proposed method. Step 1 is the input of a sequence into the model. In step 2, base-pairing probabilities (bpp) are calculated using the McCaskill algorithm based on the input sequence and a set of parameters. These parameters are subjected to optimization. In step 3, the loss is computed as the difference between the calculated base-pairing probabilities and the GT base-pairing probabilities. In step 4, the parameters are adjusted using this loss information. By using multiple RNA sequences and their corresponding GT base-pairing probabilities, steps 2 through 4 are repeated to optimize the parameters. In the figure, “Bppm” denotes the base-pairing probability matrix.

In deep learning, various predictions are performed from weights (parameters) and networks (models). During training, parameters are optimized from ground truth (GT) data using gradient descent. This relationship between parameters and models can also be applied to RNA secondary structure prediction and base-pairing probability calculations. That is, base-pairing probabilities are predicted from substructure energies (parameters) and the McCaskill algorithm (model). Accordingly, we hypothesized that if GT base-pairing probability information is available, energy parameters can be estimated by gradient descent. In other words, by differentiating the base-pairing probabilities computed by the McCaskill algorithm with respect to energy parameters, it should be possible to compute the gradient of these parameters, and from those gradients, it should be possible to optimize the parameters. In this paper, we propose a method to solve the inverse problem of estimating energy parameters using gradient descent when base-pairing probabilities are known. An overview of our method is provided in Fig. 1, which shows how the energy parameters are updated by comparing the base-pairing probabilities with the GT probabilities. This parameter update is performed using gradient descent. In the “Materials and methods” section, we present the formula for differentiating the base-pairing probabilities computed by the McCaskill algorithm with respect to energy parameters. The partition functions initially computed for estimating base-pairing probabilities can be reused in many parts of the gradient computation. In other words, the results obtained during the forward pass (for base-pairing probability calculation) can be efficiently reused during the backward pass (for parameter optimization). Additionally, each differentiated partition function can be efficiently computed using dynamic programming. By applying the proposed method, it becomes possible to estimate the parameters of modified bases from base-pairing probabilities. To demonstrate this application, in the “Results” section, we show that the energy parameters of modified bases can be estimated from the base-pairing probability of RNAs containing modified bases. Additionally, since experimentally obtained base-pairing probabilities may contain noise, we also discuss the robustness of our method against such noise. Although this method requires that GT base-pairing probabilities are already experimentally obtained, methods for adjusting base-pairing probabilities from chemical probing methods such as SHAPE have already been proposed [28–31]. It is expected that these new methods, along with the accumulation of chemical probing data for modified bases, will enable accurate energy parameter estimation for modified bases.

## Materials and methods

### Differentiation of base-pairing probabilities with respect to their energy parameters

Using the McCaskill algorithm [6], the probability ${{P}_{i,j}}$ of the *i*-th and *j*-th bases forming a base pair can be obtained as shown in the following equation.


\begin{eqnarray*}
{{P}_{i,j}} = Z_{i,j}^b\ W_{i,j}^b/{{Z}_{1,N.}}
\end{eqnarray*}

Here, ${{Z}_{i,j}}$ is the partition function, $Z_{i,j}^b$/ $W_{i,j}^b$ are the inside/outside partition functions of base pair (*i, j*), and *N* represents the sequence length. In order to estimate the optimal parameters, which is the key objective of this study, it is necessary to differentiate the base-pairing probabilities with respect to the energy parameters, as shown in the following equation.


\begin{eqnarray*}
\partial {{P}_{i,j}} = \frac{{\left( {\partial Z_{i,j}^bW_{i,j}^b + Z_{i,j}^b\partial W_{i,j}^b} \right){{Z}_{1,N}} - \ Z_{i,j}^bW_{i,j}^b\partial {{Z}_{1,N}}}}{{{{{\left( {{{Z}_{1,N}}} \right)}}^2}}}.
\end{eqnarray*}

Here, ∂ denotes partial differentiation with respect to the parameters.

Each partition function is computed using the following McCaskill algorithm.


\begin{eqnarray*}
{{Z}_{i,j}} = 1.0 + \ \mathop \sum \limits_{i < h < j} {{Z}_{i,h}}Z_{h + 1,j}^1
\end{eqnarray*}

\begin{eqnarray*}
Z_{i,j}^1 = \ \mathop \sum \limits_{i < h \le j} Z_{i,h}^b
\end{eqnarray*}

\begin{eqnarray*}
Z_{i,j}^b &=& {{e}^{ - {\mathrm{\beta }}{{{\mathrm{f}}}_1}\left( {{\mathrm{i}},{\mathrm{j}}} \right)}} + \mathop \sum \limits_{i\ < \ h\ < \ j - 1} \mathop \sum \limits_{h\ < \ l\ < \ j}Z_{h,l}^b\,{{e}^{ - {\mathrm{\beta }}{{f}_2}\left( {i,j,h,l} \right)}} \\&+& \mathop \sum \limits_{i < h < j} Z_{i + 1,h - 1}^m\,Z_{h,j - 1}^{m1}{{e}^{ - {\mathrm{\beta }}{{f}_3}}}
\end{eqnarray*}

\begin{eqnarray*}
Z_{i,j}^m = \mathop \sum \limits_{i < h \le j} \left( {{{e}^{ - {\mathrm{\beta }}{{f}_4}}} + Z_{i,h - 1}^m} \right)Z_{h,j}^{m1}\,{{e}^{ - {\mathrm{\beta }}{{f}_5}}}
\end{eqnarray*}

\begin{eqnarray*}
Z_{i,j}^{m1} = \mathop \sum \limits_{i < h \le j} Z_{i,h}^b{{e}^{ - \beta {{f}_4}}}.
\end{eqnarray*}

Here, *f*_1_​ represents the energy of a hairpin loop; *f*_2_ represents the energy of bulges, internal loops, and stacks; *f*_3_ represents the energy for forming a multiloop; *f*_4_ represents the energy proportional to the loop size within a multiloop; and *f*_5_ represents the energy per additional branch in a multiloop. Differentiating these partition functions with respect to the energy parameters yields the following equations.


\begin{eqnarray*}
\partial {{Z}_{i,j}} = \sum\limits_{i < h < j} {\partial {{Z}_{i,h}}Z_{h + 1,j}^1 + } \sum\limits_{i < {\mathrm{h}} < j} {{{Z}_{i,h}}\,\partial Z_{h + 1,j}^1}
\end{eqnarray*}

\begin{eqnarray*}
\partial Z_{i,j}^1 = \ \mathop \sum \limits_{i < h \le j} \partial Z_{i,h}^b
\end{eqnarray*}

\begin{eqnarray*}
\partial Z_{i,j}^b = - {\mathrm{\beta }}\,\partial {{f}_1}\left( {i,j} \right)\,{{{\mathrm{e}}}^{ - {\mathrm{\beta }}{{{\mathrm{f}}}_1}\left( {{\mathrm{i}},{\mathrm{j}}} \right)}}
\end{eqnarray*}

\begin{eqnarray*}
&+& \sum\limits_{i < h < j - 1} {\sum\limits_{h < l < j} {\left( {\begin{array}{@{}*{1}{c}@{}} {\partial Z_{h,l}^b\,{{{\mathrm{e}}}^{ - {\mathrm{\beta }}{{{\mathrm{f}}}_2}\left( {{\mathrm{i}},{\mathrm{j}},{\mathrm{h}},{\mathrm{l}}} \right)}} + }\\ {Z_{h,l}^b\,\left( { - {\mathrm{\beta }}} \right)\partial {{f}_2}\left( {i,j,h,l} \right){{e}^{ - {\mathrm{\beta }}{{f}_2}\left( {i,j,h,l} \right)}}} \end{array}} \right)} } \\&+& \sum\limits_{i < h < j} {\left( {\begin{array}{@{}*{1}{c}@{}} {\partial Z_{i + 1,h - 1}^m\,Z_{h,j - 1}^{m1}\,{{{\mathrm{e}}}^{ - {\mathrm{\beta }}{{{\mathrm{f}}}_3}}}}\\ { + Z_{i + 1,h - 1}^m\,\partial Z_{h,j - 1}^{m1}\,{{{\mathrm{e}}}^{ - {\mathrm{\beta }}{{{\mathrm{f}}}_3}}}}\\ { + Z_{i + 1,h - 1}^m\,Z_{h,j - 1}^{m1}\,\left( { - {\mathrm{\beta }}} \right)\,\partial {{{\mathrm{f}}}_3}\,{{{\mathrm{e}}}^{ - {\mathrm{\beta }}{{{\mathrm{f}}}_3}}}} \end{array}} \right)}
\end{eqnarray*}

\begin{eqnarray*}
\partial Z_{i,j}^m = \sum\limits_{i < h < j} {\left[ {\begin{array}{@{}*{1}{c}@{}} {\left( { - {\mathrm{\beta }}\partial {{f}_4}{{e}^{ - {\mathrm{\beta }}{{f}_4}}} + \partial Z_{i,h - 1}^m} \right)Z_{h,j}^{m1}{{e}^{ - {\mathrm{\beta }}{{f}_5}}}\,}\\ { + \left( {{{e}^{ - {\mathrm{\beta }}{{f}_4}}} + Z_{i,h - 1}^m} \right)\partial Z_{h,j}^{m1}{{e}^{ - {\mathrm{\beta }}{{f}_5}}}}\\ { + \left( {{{e}^{ - {\mathrm{\beta }}{{f}_4}}} + Z_{i,h - 1}^m} \right)Z_{h,j}^{m1}\left( { - {\mathrm{\beta }}} \right)\partial {{f}_5}{{e}^{ - {\mathrm{\beta }}{{f}_5}}}} \end{array}} \right]}
\end{eqnarray*}

\begin{eqnarray*}
\partial Z_{i,j}^{m1} = \mathop \sum \limits_{{\mathrm{i}} < {\mathrm{h}} < {\mathrm{j}}} \left( {\left( { - \,{\mathrm{\beta }}} \right)\partial {{f}_4}\,{{e}^{ - {\mathrm{\beta }}{{f}_4}}}Z_{i,h}^b + \partial Z_{i,h}^b\,{{e}^{ - {\mathrm{\beta }}\,{{f}_4}}}} \right).
\end{eqnarray*}

Additionally, the outside partition function is computed as follows:


\begin{eqnarray*}
&& W_{i,j}^b = {{Z}_{1,i - 1}}{{Z}_{j + 1,N}} + \mathop \sum \limits_{0 < k < i} \mathop \sum \limits_{j < l < N} W_{k,l}^b{{e}^{ - {\mathrm{\beta }}{{f}_2}\left( {k,i,j,l} \right)}}\\&&+ \mathop \sum \limits_{0 < {\mathrm{k}} < {\mathrm{i}}} \mathop \sum \limits_{{\mathrm{j}} < {\mathrm{l}} < {\mathrm{N}}} W_{k,l}^b\,{{e}^{ - {\mathrm{\beta }}{{f}_3}}}\\&& \left[ {Z_{k + 1,i - 1}^m\,{{e}^{ - {\mathrm{\beta }}{{f}_4}\ }} + Z_{j + 1,l - 1}^m\,{{e}^{ - {\mathrm{\beta }}{{f}_4}}} + Z_{k + 1,i - 1}^mZ_{j + 1,l - 1}^m} \right].
\end{eqnarray*}

Differentiating the outside partition function with respect to the energy parameters gives the following equation:


\begin{eqnarray*}
{l}\partial W_{i,j}^b = \partial {Z_{1,i - 1}}\,{Z_{j + 1,N}} + {Z_{1,i - 1}}\,\partial {Z_{j + 1,N}}\\+ \sum\limits_{0 < {\mathrm{k}} < {\mathrm{i}}} {\sum\limits_{j < l < N} {\left[ {\partial W_{k,l}^b{e^{ - {\mathrm{\beta }}{f_2}\left( {k,i,j,l} \right)}} + W_{k,l}^b\left( { - {\mathrm{\beta }}} \right)\partial {f_2}\left( {k,i,j,l} \right){e^{ - {\mathrm{\beta }}{f_2}\left( {k,i,j,l} \right)}}} \right]} } \\+ \sum\limits_{0 < {\mathrm{k}} < {\mathrm{i}}} {\sum\limits_{j < l < N} {\left[ {\partial W_{k,l}^b\,{e^{ - {\mathrm{\beta }}{f_3}}}\,\left( {Z_{k + 1,i - 1}^m\,{e^{ - {\mathrm{\beta }}{f_4}}} + Z_{j + 1,l - 1}^m\,{e^{ - {\mathrm{\beta }}{f_4}}} + Z_{k + 1,i - 1}^mZ_{j + 1,l - 1}^m} \right)\;} \right.} } \\+ \;W_{k,l}^b( - {\mathrm{\beta }})\partial {f_3}\,{e^{ - {\mathrm{\beta }}{f_3}}}\left( {Z_{k + 1,i - 1}^m\,{e^{ - {\mathrm{\beta }}{f_4}}} + Z_{j + 1,l - 1}^m\,{e^{ - {\mathrm{\beta }}{f_4}}} + Z_{k + 1,i - 1}^mZ_{j + 1,l - 1}^m} \right)\;\;\\+ W_{k,l}^b\,{e^{ - \beta {f_3}}}\left( {\partial Z_{k + 1,j - 1}^m\,{e^{ - \beta {f_4}\;}}\; + \;Z_{k + 1,j - 1}^m\,\left( { - \beta } \right)\partial {f_4}{e^{ - \beta {f_4}}}\;} \right.\\+ \;\partial Z_{j + 1,l - 1}^m\,{e^{ - \beta {f_4}}} + Z_{j + 1,l - 1}^m\left( { - {\mathrm{\beta }}} \right)\partial {f_4}{e^{ - \beta {f_4}}}\;\\+ \left. {\partial Z_{k + 1,i - 1}^mZ_{j + 1,l - 1}^m\; + \;Z_{k + 1,i - 1}^m\partial Z_{j + 1,l - 1}^m} \right).
\end{eqnarray*}

Among the energy parameters, *f*_3_​, *f*_4_​, and *f*_5_ are usually treated as constants independent of the base types, and since the derivative of a constant is zero, this property can be utilized to reduce the computational cost. Furthermore, the differentiation of each partition function can be efficiently computed using the same dynamic programming approach as in the original partition function calculation. Additionally, the partition functions that appear in the differentiated equations can be efficiently computed by reusing the results obtained during the calculation of base-pairing probabilities. In recent studies in which the McCaskill algorithm has been automatically differentiated with respect to sequence profiles or parameters, JAX has been carefully used for implementation, considering memory usage and computational time [32, 33]. In our method, it is sufficient to prepare a dynamic programming table specifically for the differentiation of the partition function. As an example of computational time, for a case in which 15 parameters are optimized using 135 sequences of 50 bases each, the GT values converge after 300 epochs (40 500 updates) (see the “Results” section). This computation was completed in ~2 h using a single core of an Intel Core i7-13800H.

Typically, techniques such as logsumexp or scaling are used in partition function calculations to prevent overflow. However, since the sequence length in this study was 150 nucleotides or fewer bases, these techniques were not applied in the initial version of this method. In future versions, an implementation using scaling will be made available.

### Implementation of the energy parameter optimization method using gradient descent

The tool for optimizing energy parameters was newly implemented based on RintC [34], which we previously developed. We also utilized RintC for the calculation of base-pairing probabilities with parameters during optimization. Similar to RintC, the implementation was written in C++ and compiled using g++ (GCC version 11.4.0). The new implementation is publicly available on GitHub.

The initial value of the energy parameters for the modified bases under optimization was set to −1.00 kcal/mol. As the loss function, we used the absolute error between the marginalized base-pairing probabilities calculated with GT parameters and those with estimated parameters (see below).


\begin{eqnarray*}
\textit{loss} = \mathop \sum \limits_i \left| {\textit{MBPP}_i^{GT} - MBP{{P}_i}} \right|.
\end{eqnarray*}

Here, $MBPP_i $ is the marginalized base-pairing probability, that is $MBP{{P}_i} = \mathop \sum \limits_j BP{{P}_{i,j}}.$ Instead of using the full base-pairing probability matrix, we used the marginalized base-pairing probabilities because we considered that this approach would reduce the impact of noise affecting each individual nucleotide position. Furthermore, our method is expected to be used with chemical probing data. Chemical probing experiments are considered to measure the degree of solvent exposure of a given base *i*, rather than the probability that base *i* pairs with a specific base *j*. Therefore, we considered that using the marginalized base-pairing probability, which represents the probability that base *i* pairs with any other base, would be well suited to this approach.

The gradient was computed using the differentiated equations of the energy parameters, and updates were iteratively performed to minimize the loss function. Multiple learning rates were considered, and a final learning rate of 50.0 was ultimately used. The program allows the learning rate to be configured through a command-line option.

### Calculation of base-pairing probabilities and addition of noise

To compute the GT base-pairing probabilities using the currently known m^6^A parameters determined by Kierzek *et al.* [12], RintC was used.

In order to add noise to the base-pairing probabilities, we used the NumPy library in Python to generate noise according to a normal distribution. The generated noise was then added to the base-pairing probabilities ${{P}_{i,j}}$ calculated by RintC, following the equation below.


\begin{eqnarray*}
P_{i,j}^{{\mathrm{new}}} = {{P}_{i,j}}\left( {1 + {{{\mathrm{\xi }}}_{i,j}}} \right),{\mathrm{\ }}{{{\mathrm{\xi }}}_{i,j}} \sim \mathcal{N}\left( {0,{{{\mathrm{\sigma }}}^2}} \right).
\end{eqnarray*}

Base-pairing probabilities were clipped to zero if noise caused them to become negative.

### Evaluation of estimated energy parameters

To evaluate the estimated energy parameters, we used them to calculate base-pairing probabilities and the associated MFE structures. The GT parameters were based on the stacking energy parameters determined by Kierzek *et al.* [12]. For the calculation of base-pairing probabilities and MFE structures, we used RNAfold (version 2.4.6) ([20], [35]). Since both RNAfold and our RintC implementation are based on the nearest-neighbor model and use the same parameter set, the MFE structures predicted by RNAfold can be used to evaluate the parameters estimated by our method.

Root mean squared deviations (RMSDs) were used to compare base-pairing probabilities. Recall and precision were used to compare MFE structures. Recall is the proportion of base pairs predicted using the GT parameters that were also predicted using the parameters obtained through inverse calculation. Precision is the proportion of base pairs predicted using the parameters estimated by our method.

## Results

### Estimation of modified base energy parameters using gradient descent

To verify whether the implementation described in the “Materials and methods” section can be used to estimate the energy parameters of modified bases, we prepared sequences containing modified bases along with the corresponding GT base-pairing probabilities. For the modified bases, we used the m^6^A stacking parameters determined by Kierzek *et al.* [12]. They determined m^6^A substructure parameters through optical melting experiments. In the context of RNA secondary structure prediction, there are 15 nearest-neighbor stacking parameters that involve m^6^A-modified bases. Among canonical base pairs, the six commonly considered pairs are GC, CG, AU, UA, GU, and UG. With m^6^A modification, two additional base pairs are introduced: m^6^AU and Um^6^A. For stacking interactions between canonical and modified base pairs, there are 6 × 2 = 12 possible combinations. In addition, stacking interactions between two modified base pairs yield three unique combinations when considering symmetry. Taken together, this results in a total of 15 distinct nearest-neighbor parameters that involve m6A modifications. The report by Kierzek *et al.* included parameters for all 15 possible adenine stacking configurations within the nearest-neighbor model, noting an average error of ± 0.3 kcal/mol. Although some degree of error exists, chemical probing and NMR results for m^6^A-modified RNAs, which are known to alter protein binding affinity, show good agreement with calculations using their m^6^A parameter set. Furthermore, transcriptome-scale PERS experimental results also support the validity of the determined parameters [12]. Consequently, these parameters have been incorporated into widely used RNA structure prediction packages such as RNAstructure [36] and ViennaRNA [20], making them broadly utilized.

**Figure 2.**
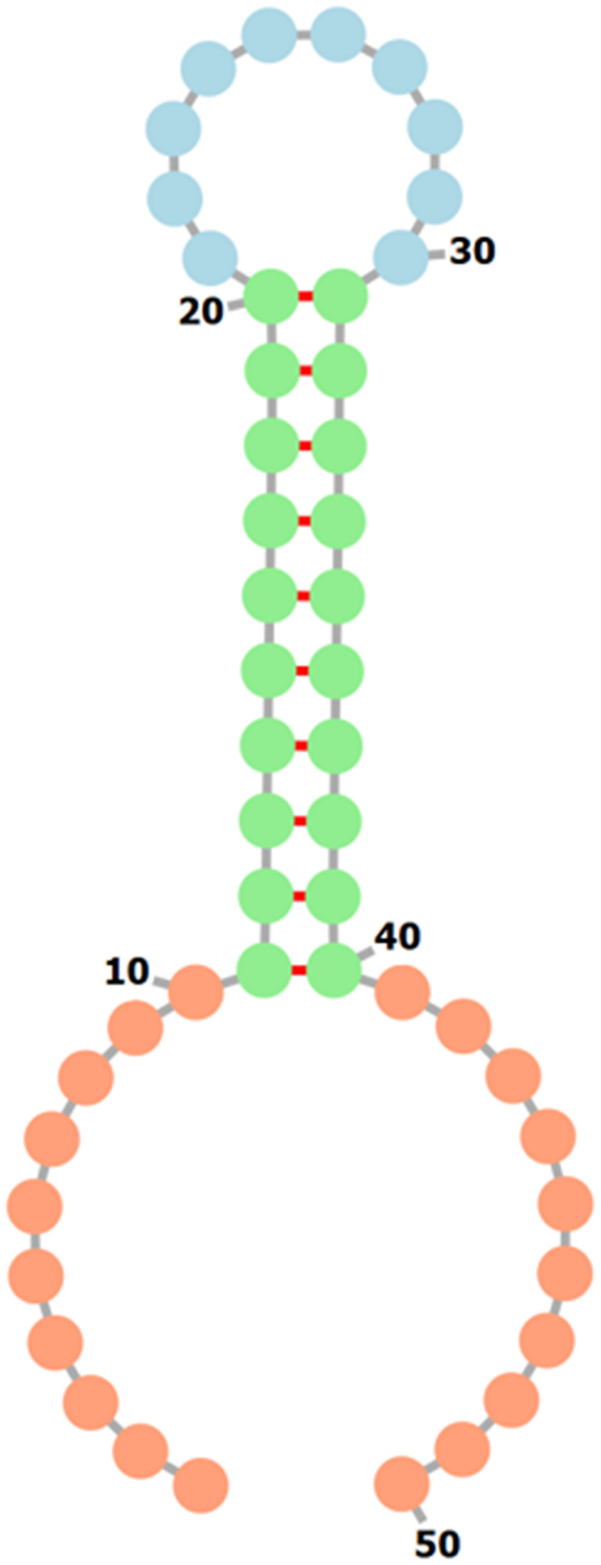
Example of the generated 50-base secondary structure. The 5′ and 3′ ends each consist of 10 random bases. Bases 11 to 20 (from the 5′ end) and bases 31 to 40 are designed as complementary sequences. These sequences contain a modified base and are generated to include one stack as defined by the stacking parameters. For each stack, there are nine design patterns, such that the position of the stack is shifted one base downstream, ranging from being included in bases 11–12 to being included in bases 19–20. Since 15 types of stacks are defined, the minimum set consists of 135 sequences. Bases 21 to 30 are random sequences.

**Figure 3.**
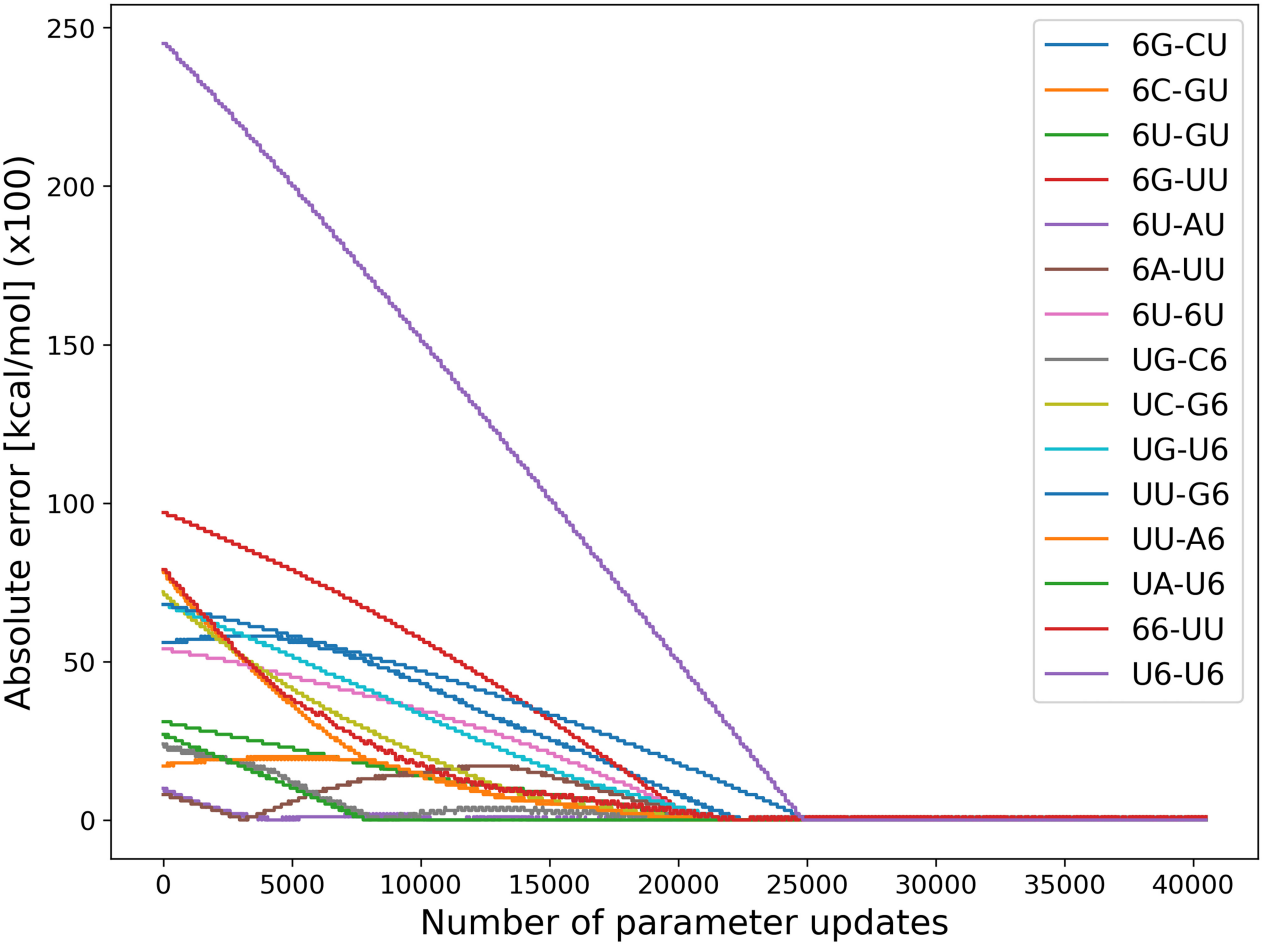
Transition of the error between each stacking parameter update and the GT parameter. The vertical axis shows the absolute value of the difference between the GT parameters and the parameters during optimization. The horizontal axis shows the number of updates. Since there were 135 sequences and the optimization was run for 300 epochs, a total of 40 500 updates were performed.

In our experiment, the generated sequences, including m^6^A, were designed to be 50 nucleotides long. The 5′ and 3′ ends each contained 10 random nucleotides, the inner 10 nucleotides on both sides formed a stem, and the central 10 nucleotides were also randomly generated (Fig. 2). The stem-forming regions included m^6^A modifications and were designed such that stacks defined by stacking parameters were present within the stem. A total of 15 types of stacking parameters were defined (Table 1), and the sequences were designed so that each stack appeared at a specific position within the stem. The complete sequences are provided in the Supplementary data. The GT base-pairing probabilities were calculated using RintC with the energy parameters of the modified bases. Using the designed sequences and their base-pairing probabilities, we applied our gradient descent-based parameter estimation tool to verify whether the energy parameters of the modified bases used in the calculations could be reproduced. The difference between the estimated and GT energy parameters over epochs of gradient descent is shown in Fig. 3. Fifteen types of stacking parameters were defined for sequences containing m^6^A, and Fig. 3 shows that the loss for each stacking parameter decreased with each update. The estimated energy parameters converged to the GT values after ~300 epochs (Table 1). This result demonstrates that, given the base-pairing probabilities, the GT energy parameters can be almost completely reconstructed.

**Table 1.** GT and the estimated parameters for each stacking

Stacking	6CUG	UC6G	6GUC	UG6C	6UUA	6AUU	UU6A	UA6U	6UUG	6UU6	UG6U	UU6G	66UU	6GUU	U66U
GT	−1.79	−1.72	−1.56	−1.24	−1.1	−0.92	−0.83	−0.73	−0.69	−0.46	−0.32	−0.32	−0.21	−0.03	1.45
Optimized	−1.79	−1.72	−1.56	−1.25	−1.1	−0.92	−0.83	−0.73	−0.69	−0.46	−0.32	−0.32	−0.22	−0.03	1.45

Energy values are presented as kcal/mol. The GT row shows the energy parameters used for calculating the base-pairing probabilities. The optimized row shows the energy parameters optimized after 300 epochs of computation.

### Estimating energy parameters from base-pairing probabilities containing noise

**Figure 4.**
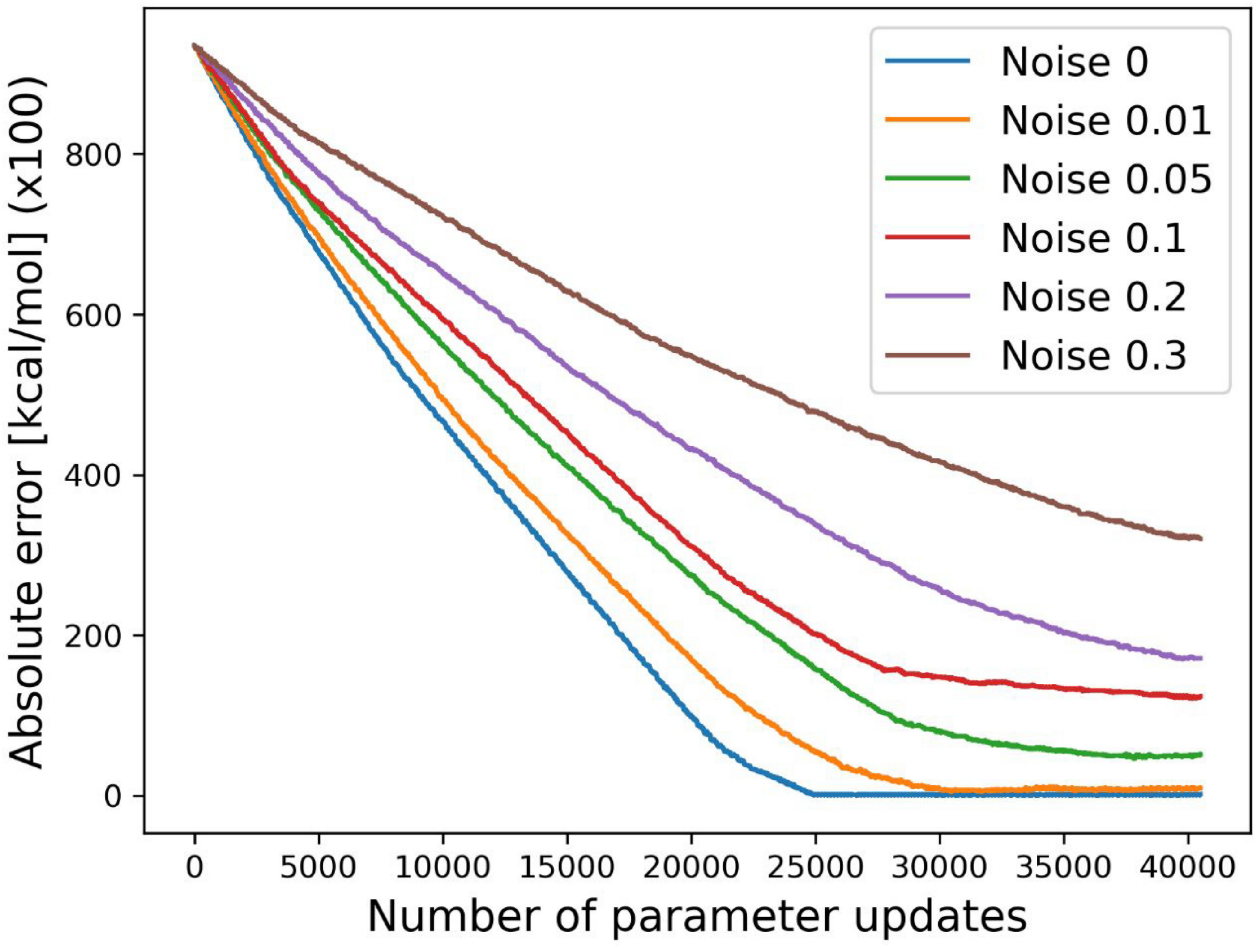
Convergence of energy parameters when random noise following a normal distribution was added to the base-pairing probabilities. The absolute sum of the differences between the GT parameters and the estimated parameters was used as the error, and the change in error was plotted after each parameter update. The vertical axis represents the error. The horizontal axis represents the number of updates. Since there were 135 sequences and the optimization was run for 300 epochs, a total of 40 500 updates were performed. Noise was introduced with standard deviations ranging from 0 (i.e. no noise) to 0.3.

The method proposed in this study optimizes energy parameters from the marginalized base-pairing probabilities. When base-pairing probabilities are obtained through experiments such as SHAPE, it is expected that experimental noise will be included. Therefore, the evaluation of the robustness of our method against noise is important in practice. We added random noise to the GT base-pairing probabilities and examined how the convergence of energy parameters changed depending on the magnitude of the noise. We assumed that the noise follows a normal distribution and considered its standard deviation as the noise level (see the “Materials and methods” section). Figure 4 shows the results of adding noise with standard deviations ranging from 0.01 to 0.3. Fifteen types of stacks containing m^6^A were defined, and the loss was computed as the sum of absolute differences between GT and estimated energy parameters across all 15 stacking parameters. Table 2 shows the convergence of the total absolute error for each dataset with different levels of noise added, across a range of standard deviations. In the experiment in which the m^6^A stacking parameters were determined, there was an experimental error of ±0.3 kcal/mol [12], and even with the addition of noise with a standard deviation of 0.3, only 3 out of 15 estimated parameters differed from the GT parameters by more than ±0.3 kcal/mol (Table 2).

**Table 2.** Absolute differences between the parameter values after 300 epochs and the GT parameters when noise was added to the base-pairing probabilities

Stacking	6CUG	UC6G	6GUC	UG6C	6UUA	6AUU	UU6A	UA6U	6UUG	6UU6	UG6U	UU6G	66UU	6GUU	U66U
Noise 0	0	0	0	0	0	0	0	0	0	0	0	0	0.01	0	0
Noise 0.01	0.01	0	0.01	0.01	0	0	0.01	0.01	0.02	0.01	0	0.01	0	0	0
Noise 0.05	0.01	0.01	0.05	0	0.01	0	0.01	0.02	0.2	0.04	0.03	0.03	0.04	0.01	0.05
Noise 0.1	0.01	0.03	0.2	0.01	0.07	0.06	0.13	0.05	0.13	0.26	0.04	0.03	0.07	0.08	0.07
Noise 0.2	0.12	0.11	0.07	0.17	0.12	0.01	0.24	0.1	0.02	0.01	0.04	0.09	0.26	0.04	0.31*
Noise 0.3	0.11	0.17	0.28	0.03	0.37*	0.19	0.56*	0.51*	0.23	0.1	0.16	0.21	0.21	0.03	0.04

The noise 0 row shows results from the case in which no noise was added. The noise 0.01–0.3 rows show results from cases in which the standard deviation of the added noise to the base-pairing probabilities varied across this range. Asterisks indicate the cells in which the difference from the GT parameters exceeded 0.3 kcal/mol.

### Evaluation of parameter estimation performance using sequences with multiple modifications

We performed energy parameter estimation from base-pairing probabilities containing noise; however, the data used in the previous experiments included only 1–2 modified nucleotides per 50-nucleotide sequence. When the number of modified bases is higher, the parameter set involving those modifications is expected to be used more frequently, thereby increasing the opportunity for parameter updates. To test this, we conducted an evaluation using random sequences with an increased number of modifications. Specifically, we generated 150-nucleotide sequences in which eight adenosines were modified to m^6^A and performed parameter estimation under this condition (Supplementary Fig. S1). Together with previous results, the method showed good convergence under all noise levels after 40 500 update steps. This suggests that sequences containing a greater number of modified bases are more robust to noise in parameter estimation.

Recent mRNA vaccines contain pseudouridine modifications at all uridine positions. To assess whether our method can correctly estimate parameters even when all potentially modifiable sites are actually modified, we tested sequences in which all adenosines were replaced with m^6^A (Supplementary Fig. S2). Although the parameters containing adenosine remained at their initial values and were not updated, the other parameters successfully converged to their correct values. In cases such as mRNA vaccines, where all target sites in the molecule are modified, parameters corresponding to the unmodified target base are not required for structure prediction. Therefore, these results indicate that the method remains practical and applicable under such fully modified conditions.

### Evaluation of estimated energy parameters

In this section, we present the results of evaluating the estimated energy parameters by calculating secondary structures and base-pairing probabilities using both the estimated and GT energy parameters. The estimated parameters used for evaluation here are the worst-performing ones from our experiments, derived from cases containing only 1 to 2 modified bases out of 50.

**Figure 5.**
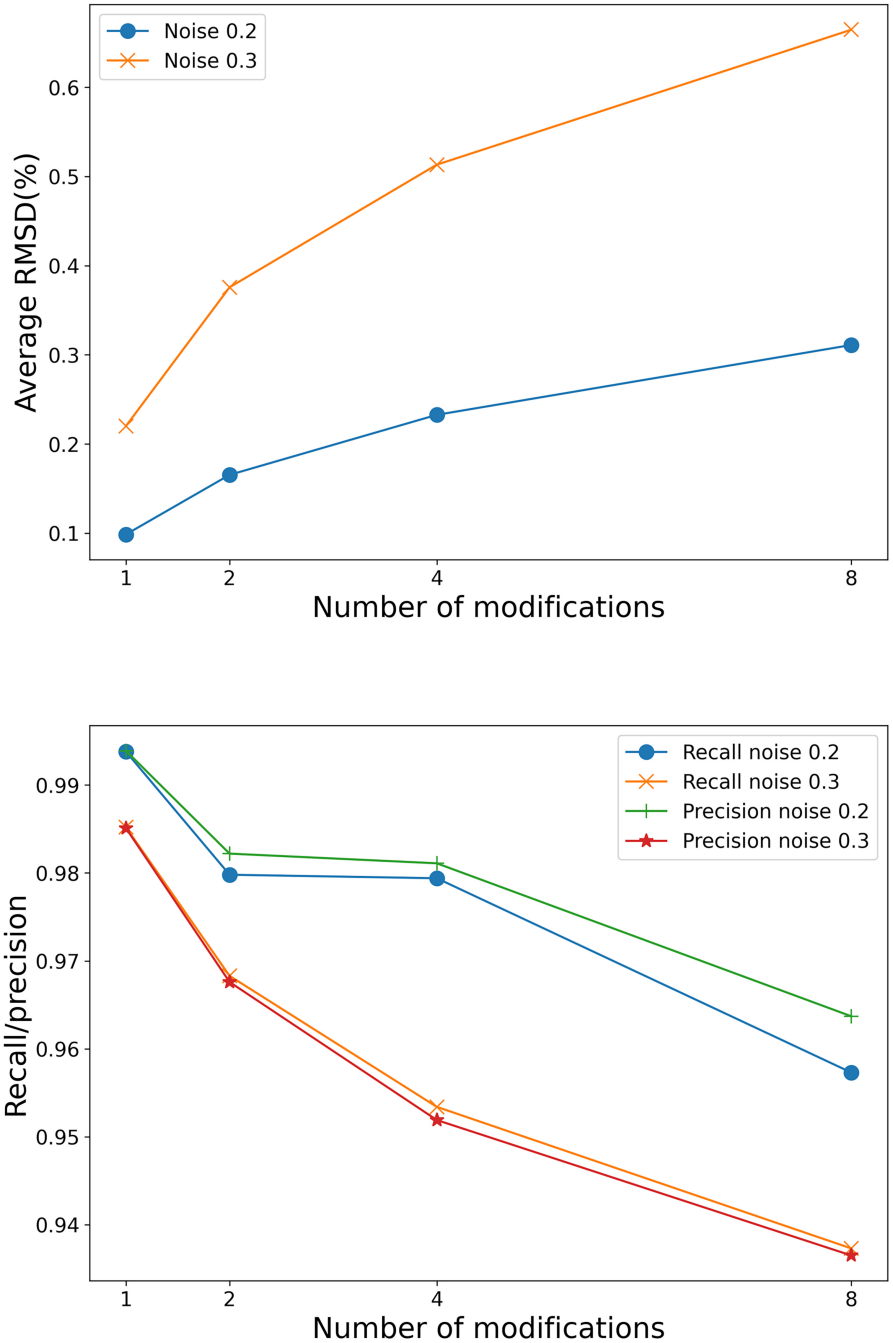
Base-pairing probabilities and MFE structures were calculated using the modified base energy parameters estimated from inverse calculations based on noisy base-pairing probabilities. These results were then compared with the base-pairing probabilities and MFE structures computed using the experimentally determined energy parameters of modified bases reported by Kierzek *et al.* [12]. RMSD was used to compare base-pairing probabilities (top panel). Recall and precision were used to compare MFE structures (bottom panel). In both graphs, the horizontal axis represents the number of modified bases present in the 50-nucleotide random sequences.

As shown earlier, the energy parameters can be reasonably well recovered even when noise is added to the base-pairing probabilities. However, it remains unclear how much small deviations in energy parameters affect secondary structure prediction. Thus, we investigated how parameters obtained under noisy conditions affect the accuracy of secondary structure prediction. Several studies have already investigated the impact of variation in energy parameters on RNA secondary structure prediction [37–39]. It has been shown that when perturbations are introduced to energy parameters, the MFE structures can change significantly, whereas the accuracy of base-pairing probabilities remains robust [37, 38]. To evaluate the robustness of the estimated energy parameters for RNA secondary structure prediction, we computed the MFE structures and base-pairing probabilities using those parameters. The nucleotide sequences used for the calculations were random 50-base-long sequences designed to include modified bases (m^6^A) at random positions in groups of 1, 2, 4, or 8 bases, respectively. The differences between the base-pairing probabilities and MFE structures calculated using the experimentally determined m^6^A stacking parameters [12] and those estimated by our method are shown in Fig. 5 and Table 3. For evaluating the differences, we used RMSD values for base-pairing probabilities and precision and recall for MFE structure differences, consistent with Zuber *et al.* [39] (see the “Materials and methods” section). Even when noise with a standard deviation of 0.3 was added to the GT base-pairing probability data, the estimated energy parameters yielded more-robust MFE structures and base-pairing probabilities compared with the results of Zuber *et al.*, who sampled experimental values within the range of experimental error (3σ) (RMSD 1.87% ± 0.40%, recall 89.60% ± 2.13%, precision 89.19% ± 2.21%) [39]. It should be noted, however, that the nucleotide sequences used by Zuber *et al.* did not include modified bases, meaning that the comparisons were not performed on identical sequences.

**Table 3.** Comparison of the results obtained using energy parameters inversely calculated from noisy base-pairing probabilities with the results obtained using the GT energy parameters for computing base-pairing probabilities of RMSD and MFE structures (recall and precision)

	random 1000 (STD = 0.2, 8/50 modified)	random 1000 (STD = 0.3, 8/50 modified)
Mean RMSD	0.31% ± 0.28%	0.66% ± 0.62%
Mean recall	0.96 ± 0.17	0.94 ± 0.20
Mean precision	0.96 ± 0.16	0.94 ± 0.20
	stem loop 1080 (STD = 0.2)	stem loop 1080 (STD = 0.3)
Mean RMSD	0.08% ± 0.18%	0.14% ± 0.29%
Mean recall	0.99 ± 0.03	0.99 ± 0.04
Mean precision	0.99 ± 0.03	0.99 ± 0.04

The top panel shows the results for completely random 50-nucleotide sequences with eight modified bases. The bottom panel shows the results for sequences in which the stacking interactions of modified bases were positioned within a stem-loop structure, as shown in Fig. 2. In each panel, the left side presents the case in which the standard deviation of the noise added to the base-pairing probabilities was 0.2, and the right side presents the case in which the standard deviation was 0.3.

In addition to the above evaluation, to test conditions favorable for base-pair formation involving modified bases, we generated sequences that guarantee the presence of stacking interactions defined by the stacking parameters (Fig. 2 and Supplementary data). For these sequences, we calculated base-pairing probabilities and MFE structures using both the estimated parameters and the reference parameters experimentally determined by Kierzek *et al.* [12] and then compared the results (Fig. 5, Table 3, and Supplementary Fig. S3). Our results were more robust than those of Zuber *et al.*, as was also observed in the evaluation described earlier.

Zuber *et al.* reported that introducing noise into the experimental data used for determining energy parameters induced correlations among the estimated parameters [39]. Similarly, in our study, we observed correlations between certain parameters under varying levels of noise (Supplementary Fig. S4 and Supplementary Table S1). For example, when the 6U–UA stack was estimated to have a higher value than the GT energy parameter, the 6C–UG stack tended to be estimated at a lower value than the GT value (Supplementary Table S1). This correlation is further examined in the “Discussion” section.

## Discussion

In this study, we developed and evaluated a method for calculating energy parameters from base-pairing probabilities. Several approaches have been developed to compute experimentally adjusted base-pairing probabilities using chemical probing techniques, such as the SHAPE method [28–31]. RNAfold implements three approaches for incorporating chemical probing data [20]. Among them, the method by Deigan *et al.* adjusts the pseudo-energies of nucleotides involved in base pairing [30]; Zarringhalam *et al.* proposed a method that modifies the pseudo-energies of loop-containing structures [29]; and Washietl *et al.* introduced a method of reflecting reactivity data by maximizing the consistency of energy parameters of substructures with experimental data [28]. For detailed descriptions and comparisons of these methods, we refer the reader to the review article [40]. Each of these methods involves parameters, such as the extent to which chemical reactivity is translated into energetic penalties or bonuses. Determining the optimal method and corresponding parameter values is likely to be non-trivial. This may present an opportunity for our proposed framework to enhance the utility of chemical probing data.

Currently, chemical probing data at the transcriptome scale have been published. These datasets are expected to contain a mixture of chemical probing results from both mature and immature RNA molecules. In other words, the probing results may reflect a heterogenous population of molecules, some of which carry endogenous RNA modifications, while others do not. Therefore, the currently available data may be insufficient for estimating the energy parameters of modified bases. However, in mRNA vaccines, all uridines are modified to pseudouridine, and data in which all specific sites are modified are thought to be obtainable. In our results, we successfully estimated the parameters for m^6^A when all adenosines were modified (Supplementary Fig. S2). These results may suggest that, once such fully modified data become available, our method can be utilized to estimate the energy parameters of modified bases.

Currently available energy parameters for modified bases have been determined primarily for stacking parameters. While the present study also focused on estimating stacking parameters, it should also be possible to estimate parameters for bulges, internal loops, and hairpin loops. If these parameters can indeed be accurately determined, further improvements in both the prediction accuracy of RNA structures containing modified bases and the design of mRNA would be expected.

We observed correlations among the estimated parameters (Supplementary Fig. S4 and Supplementary Table S1). Zuber *et al.* demonstrated that energy parameters estimated from experimental data with added noise lead to more robust structure predictions than those obtained by directly adding noise to the energy parameters themselves [39]. This increased robustness is thought to arise from the correlations that emerge among the estimated parameters as a consequence of the addition of noise to the experimental data. If the correlations between parameters observed in our method have an effect similar to those reported by Zuber *et al.* for noisy experimental data, then the energy parameters estimated by our method may also produce robust RNA structure prediction.

With the advancement of deep learning, several libraries, such as PyTorch and JAX, have been developed that support automatic differentiation using GPUs. These libraries enable automatic differentiation by simply implementing their functions, eliminating the need to manually derive derivative expressions. However, since the computational graph must be retained during the parameter estimation process, memory usage tends to be high. Typically, general-purpose software is associated with greater computational and memory demands, while domain-specific software tends to be more efficient in terms of both resources. In this study, we developed a specialized tool that analytically computes the derivatives of base-pairing probabilities. As a result, unnecessary computations and intermediate storage are minimized, allowing parameter estimation and functional testing to be performed even on standard laptop computers without the need for GPU acceleration.

The method employed here is gradient descent, a technique commonly used in deep learning. Accordingly, numerous strategies for handling noisy data have been developed [41], and the potential to incorporate these is a significant advantage. As more experimental data become publicly available, further refinements can be made to adapt to the specific noise characteristics of data. We believe this method will provide a practical approach for estimating energy parameters.

## Supplementary Material

lqaf171_Supplemental_Files

## Data Availability

The data underlying this article are available in the supplementary data. The software developed and used in this study is available at the following GitHub repository, https://github.com/yamamu-kaz/rintc_grad, and in Zenodo, https://doi.org/10.5281/zenodo.15532332.
