# Supplementary material for: A method for estimating energy parameters of RNAs by differentiating base-pairing probabilities
Source: NAR Genom Bioinform. 2025 Dec 8;7(4):lqaf171. doi: 10.1093/nargab/lqaf171 (PMC12684387; doi:10.1093/nargab/lqaf171)
Supplement: lqaf171_Supplemental_Files [file lqaf171_supplemental_files.zip › energyparam_diff_paper_supply_E_revise1.docx]

Supplementary data


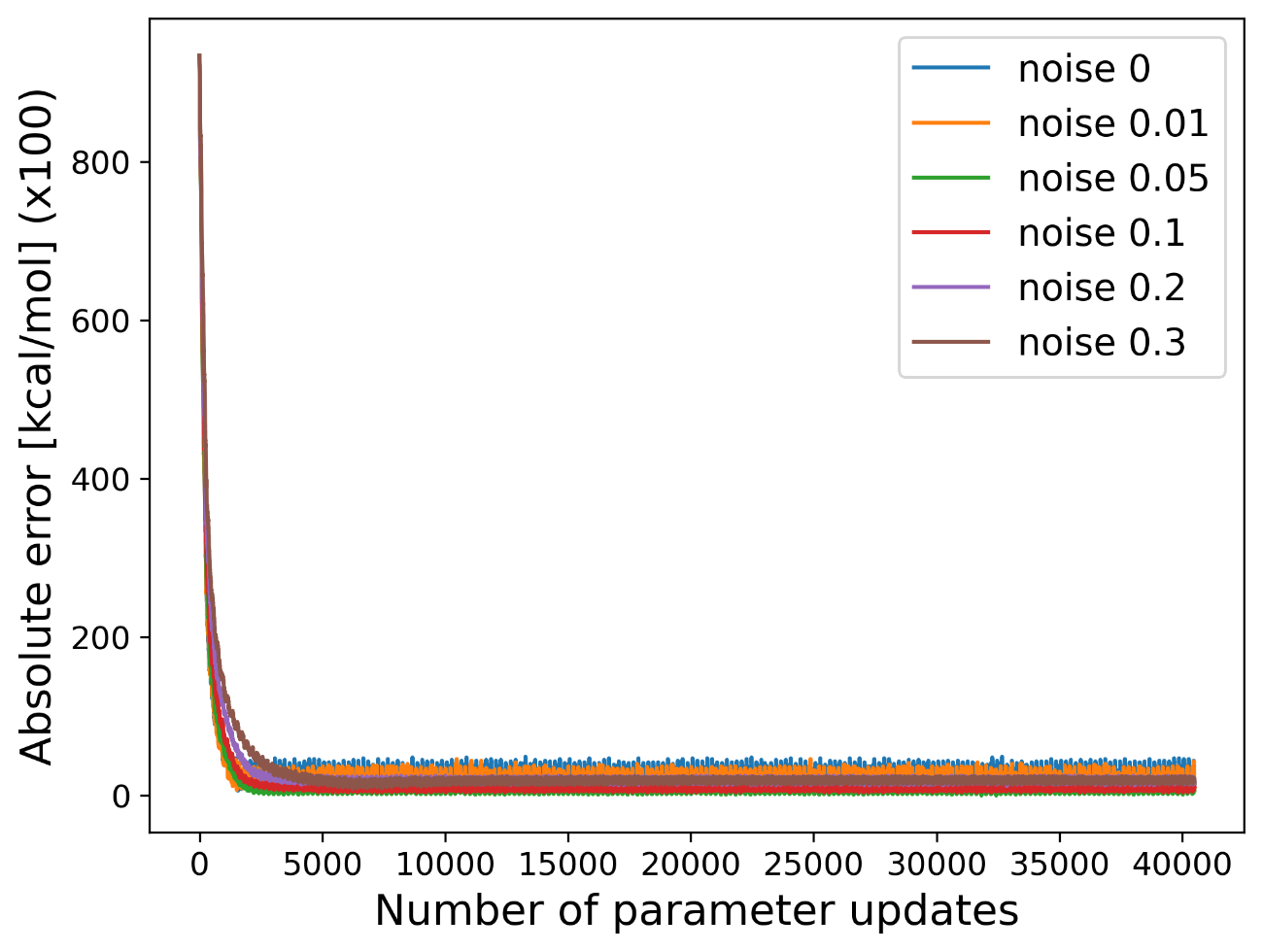


Figure S1

Convergence of energy parameters when random noise following a normal distribution was added to the base-pairing probabilities. The sequences generated for the calculations were completely random 150-nucleotide sequences, and 200 such sequences were prepared. Each RNA molecule was designed to contain eight m6A bases. The absolute sum of the differences between the ground truth (GT) parameters and the estimated parameters was used as the error, and the change in error was plotted after each parameter update. The vertical axis represents the error. The horizontal axis represents the number of updates. To compare with the results in Figure 4, the calculations were performed up to 40,500 update steps. Noise was introduced with standard deviations ranging from 0 (i.e., no noise) to 0.3.


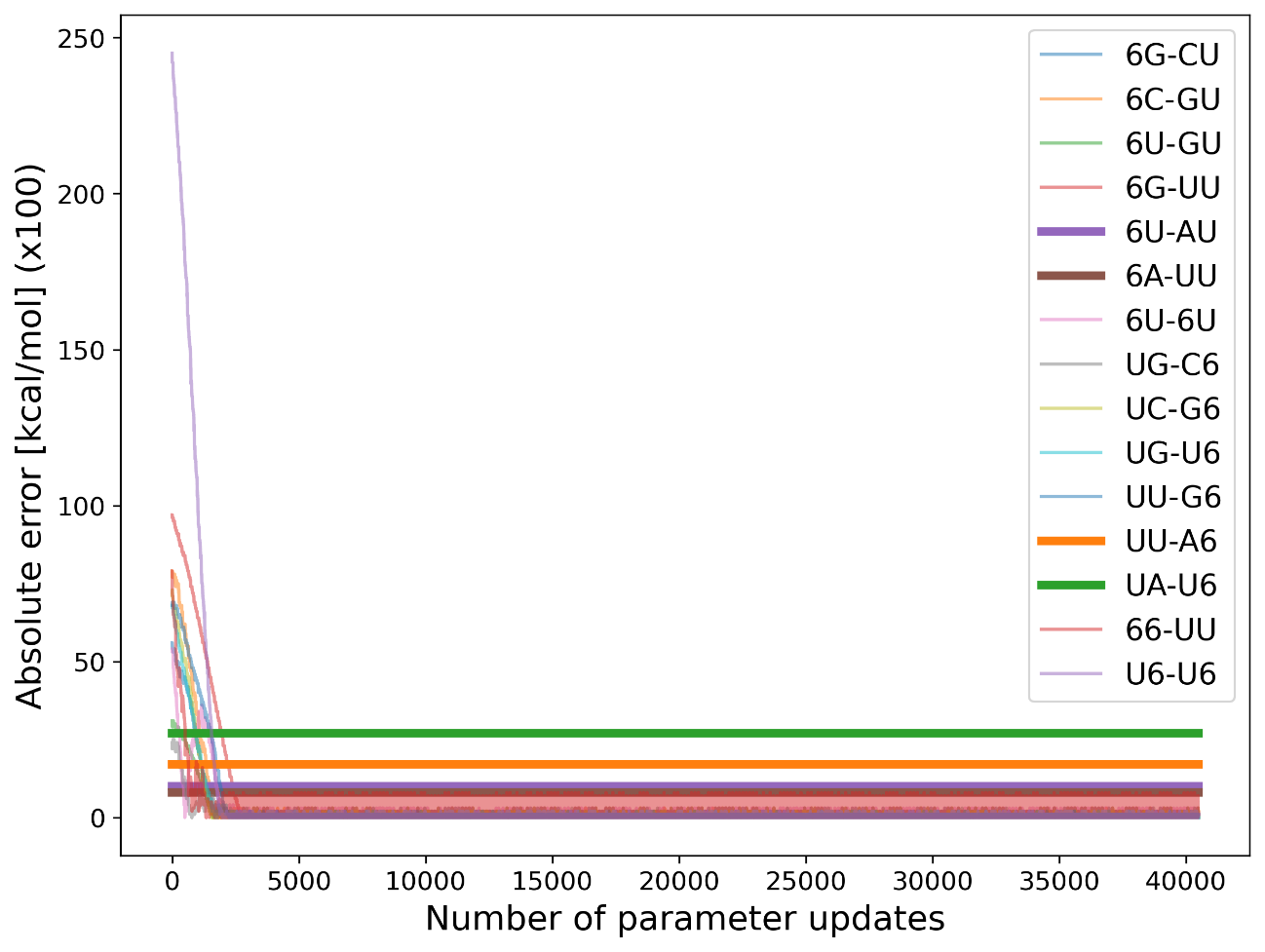


Figure S2

Transition of the error between each stacking parameter update and the ground truth (GT) parameter. The vertical axis shows the absolute value of the difference between the GT parameters and the parameters during optimization. The horizontal axis shows the number of updates. Unlike in Figure 3, where only 1–2 modified bases were included, all adenines were treated as m6A in this calculation. In the dataset used for this figure, since all adenines were modified, no stacks containing unmodified adenine were present, and thus the corresponding parameters were not updated (indicated by bold lines). To compare with the results in Figure 4, the calculations were performed up to 40,500 update steps.


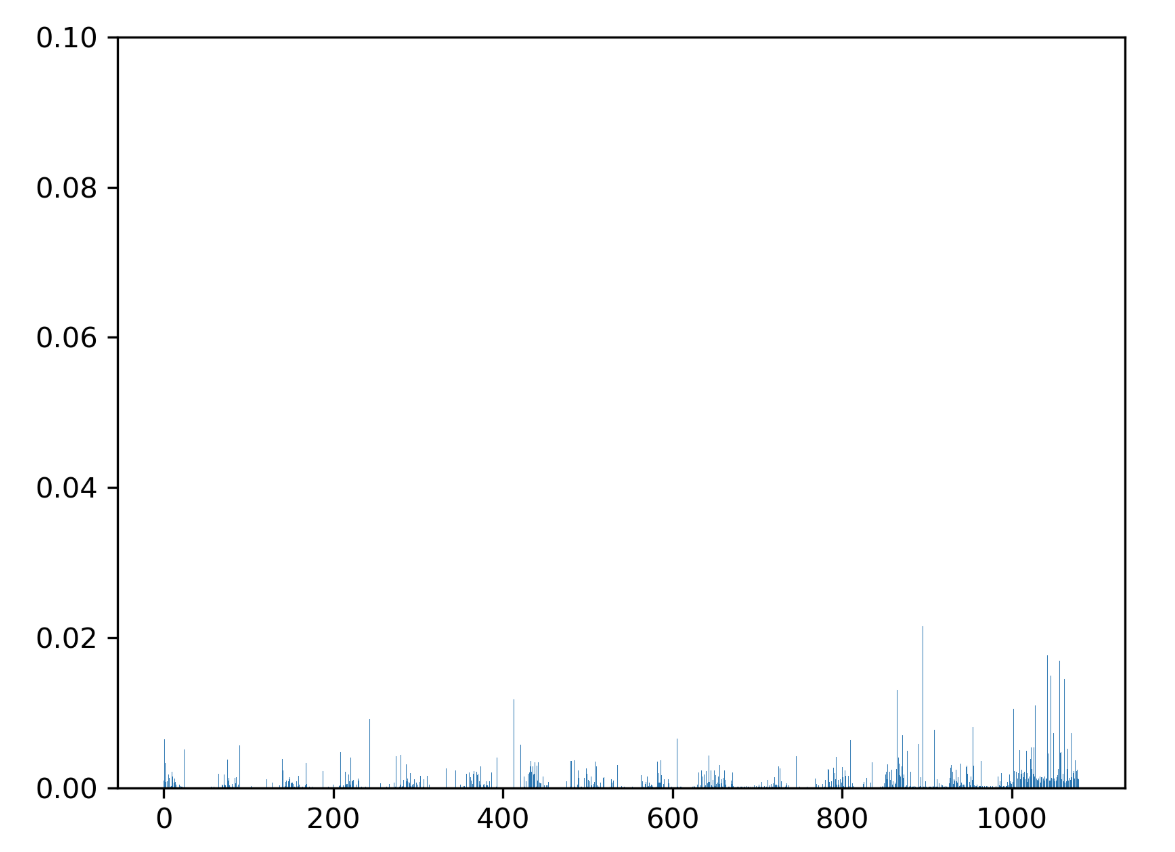


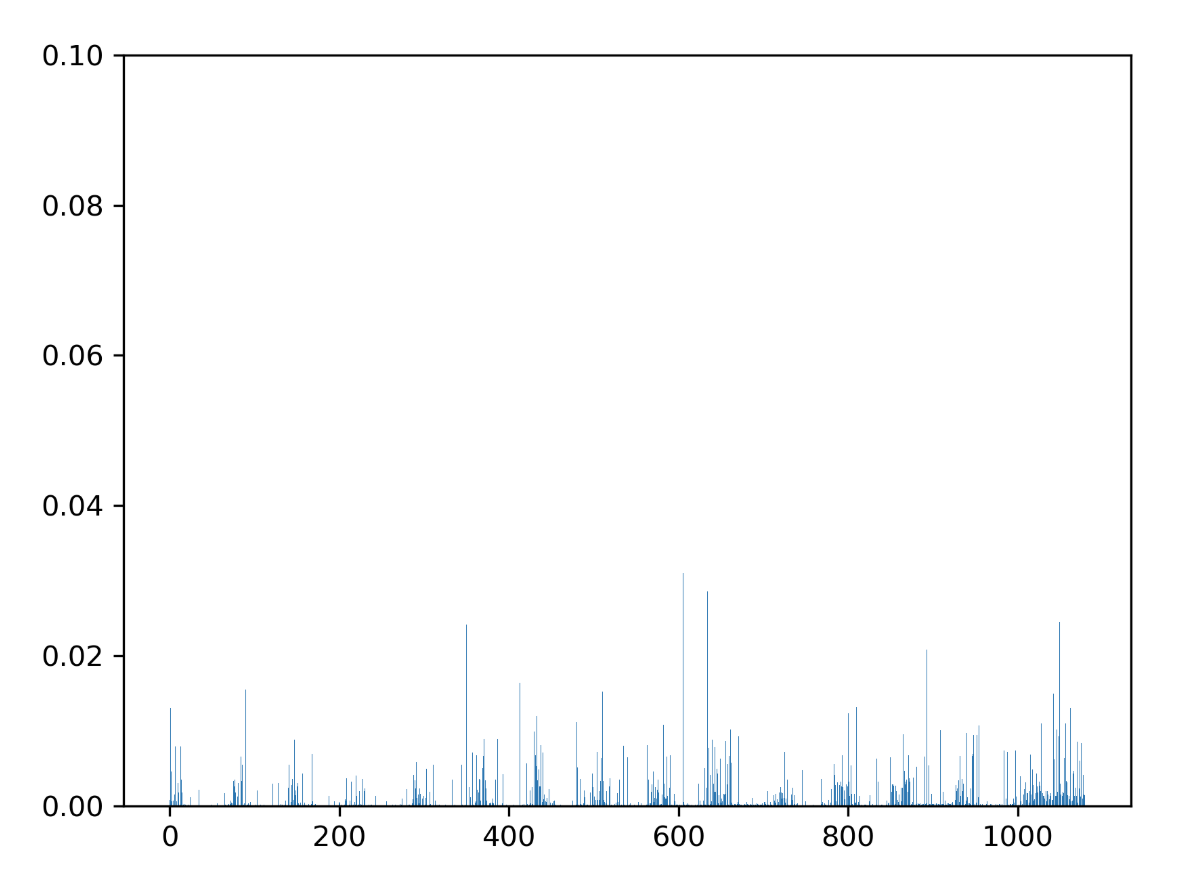


Figure S3

The base-pairing probabilities were computed using both the energy parameters inversely calculated from noisy base-pairing probabilities and the reference energy parameters, and their differences were compared using RMSD. The horizontal axis represents the sequence index (a total of 1,080 sequences). The vertical axis represents the RMSD.

The top panel shows the results when the standard deviation of the added noise in the base-pairing probabilities is 0.2. The bottom panel shows the results when the standard deviation of the added noise is 0.3.

Figure S4

The correlation matrix of the energy parameters estimated from base-pairing probabilities was computed after adding various levels of noise (standard deviation 0.20 to 0.3) . 15 types of stacking parameters for m6A are defined. The horizontal and vertical axes represent the different stacking parameters. The correlations were calculated based on the differences between the converged parameter values and the reference parameter values.

| stacking | 6C UG | UC 6G | 6G UC | UG 6C | 6U UA | 6A UU | UU 6A | UA 6U | 6U UG | 6U U6 | UG 6U | UU 6G | 66 UU | 6G UU | U6 6U |
| --- | --- | --- | --- | --- | --- | --- | --- | --- | --- | --- | --- | --- | --- | --- | --- |
| GT | -1.79 | -1.72 | -1.56 | -1.24 | -1.1 | -0.92 | -0.83 | -0.73 | -0.69 | -0.46 | -0.32 | -0.32 | -0.21 | -0.03 | 1.45 |
| noise 0.2 | -1.67 | -1.61 | -1.49 | -1.07 | -1.22 | -0.93 | -0.59 | -0.83 | -0.67 | -0.47 | -0.28 | -0.41 | -0.47 | 0.01 | 1.14 |
| noise 0.21 | -1.84 | -1.75 | -1.32 | -1.28 | -1.09 | -0.76 | -1.21 | -0.71 | -0.8 | -0.47 | -0.47 | -0.65 | -0.22 | -0.21 | 1.25 |
| noise 0.22 | -1.67 | -1.76 | -1.37 | -1.16 | -1.2 | -0.51 | -0.96 | -0.93 | -0.71 | -0.39 | -0.4 | -0.59 | -0.41 | 0.19 | 0.94 |
| noise 0.23 | -1.69 | -1.68 | -1.1 | -1.14 | -1.19 | -0.7 | -0.6 | -0.9 | -1.11 | -1.2 | -0.48 | -0.24 | -0.61 | -0.01 | 1.49 |
| noise 0.24 | -1.63 | -1.6 | -1.29 | -1.08 | -0.97 | -0.81 | -1.05 | -1 | -0.64 | -0.63 | -0.3 | -0.44 | -0.55 | -0.15 | 1.47 |
| noise 0.25 | -1.74 | -1.59 | -1.38 | -1.25 | -0.92 | -0.61 | -0.88 | -0.8 | -0.98 | -0.24 | -0.52 | -0.63 | -0.29 | 0 | 1.09 |
| noise 0.26 | -2.02 | -1.54 | -1.44 | -0.92 | -0.97 | -0.68 | -0.68 | -0.85 | -0.65 | -0.7 | -0.46 | -0.62 | -0.69 | -0.16 | 1.22 |
| noise 0.27 | -1.84 | -1.68 | -1.06 | -1.36 | -0.83 | -0.76 | -0.96 | -0.89 | -0.98 | -0.47 | -0.49 | -0.32 | -0.38 | 0.14 | 1.02 |
| noise 0.28 | -1.89 | -1.51 | -1.49 | -1.07 | -0.91 | -0.74 | -0.78 | -0.78 | -1 | -0.69 | -0.54 | -0.39 | -0.5 | -0.11 | 1.18 |
| noise 0.29 | -1.69 | -1.79 | -1.46 | -1.18 | -1.29 | -0.89 | -1.07 | -0.86 | -1.01 | -0.73 | -0.73 | -0.47 | -0.62 | 0.03 | 1.12 |
| noise 0.3 | -1.9 | -1.55 | -1.28 | -1.27 | -0.73 | -0.73 | -1.39 | -1.24 | -0.92 | -0.56 | -0.48 | -0.53 | -0.42 | 0 | 1.41 |

Table S1

This table shows the estimated energy parameters when Gaussian noise (with standard deviations ranging from 0.20 to 0.30) was added to the base-pairing probabilities. The columns represent the specific base patterns of the stacking parameters, and the rows show the estimated energy parameters corresponding to each added noise level. "GT" represents the ground truth energy parameters. Figure S4, which investigates the correlation between stacking parameters, was computed based on the data in this table. Specifically, in Figure S4, the correlation coefficients were calculated from the differences between the estimated energy parameters at each noise level shown in this table and the corresponding GT energy parameters.
